# Dietary characteristics of urban community-dwelling older adults with low muscle mass: the bunkyo health study: a cross-sectional study

**DOI:** 10.1186/s12877-024-05218-4

**Published:** 2024-07-18

**Authors:** Yukiko Muroga, Hideyoshi Kaga, Thu Hien Bui, Mari Sugimoto, Yuki Someya, Saori Kakehi, Hiroki Tabata, Hitoshi Naito, Abulaiti Abudurezake, Huicong Shi, Hikaru Otsuka, Yasuyo Yoshizawa, Ryuzo Kawamori, Hirotaka Watada, Yoshifumi Tamura

**Affiliations:** 1 Department of Sports Medicine and Sportology, Graduate School of Medicine, Juntendo University, 2-1-1 Hongo, Bunkyo-ku, Tokyo, 113-8421 Japan; 2 Department of Metabolism and Endocrinology, Graduate School of Medicine, Juntendo University, 2-1-1 Hongo, Bunkyo-ku, Tokyo, 113-8421 Japan; 3 Department of Sportology Center, Graduate School of Medicine, Juntendo University, 2-1-1 Hongo, Bunkyo-ku, Tokyo, 113-8421 Japan; 4 Department of Center for Healthy Life Expectancy, Graduate School of Medicine, Juntendo University, 2-1-1 Hongo, Bunkyo-ku, Tokyo, 113-8421 Japan; 5 Faculty of International Liberal Arts, Juntendo University, 2-1-1 Hongo, Bunkyo-ku, Tokyo, 113-8421 Japan

**Keywords:** Dietary intake, Low skeletal muscle mass, Sarcopenia, Older adults

## Abstract

**Background:**

With the aging of the population worldwide, extending healthy life expectancy is an urgent issue. Muscle mass has been reported to be associated with physical independence and longevity. This study aimed to investigate the characteristics of food intake in urban community-dwelling older adults with low muscle mass.

**Methods:**

This cross-sectional study used baseline data from the Bunkyo Health Study, which included 1618 urban community-dwelling older adults aged 65–84 years. All participants underwent measurement of body composition using bioelectrical impedance analysis and evaluation of nutrient and food intake using the brief-type self-administered diet history questionnaire. Participants were stratified by sex and divided into robust or low skeletal muscle mass index (SMI) groups according to the Asian Working Group for Sarcopenia criteria to compare differences in nutrient and food intake.

**Results:**

The mean age and body mass index were 73.1 ± 5.4 years and 22.6 ± 3.1 kg/m^2^, respectively. The prevalence of low SMI was 31.1% in men and 43.3% in women. In men, all food intake, including total energy intake, was similar between the low SMI group and the robust group. In women, the low SMI group had less total energy intake, and consumed lower amounts of energy-producing nutrients (protein, fat, and carbohydrates), but there were only small differences in the intake of specific foods.

**Conclusions:**

There were sex differences in food intake characteristics between urban community-dwelling older adults with low SMI and those who were robust. Advising women to increase their energy intake may be important in preventing muscle loss, and further research is needed in men.

## Introduction

The world’s population is aging rapidly. The proportion of individuals aged 65 and over worldwide (aging rate) is expected to rise to 18.7% by 2060, with further acceleration over the next 40 years [1]. The aging rate in Japan is particularly high, at 29.0% in 2022, and will reach 38.7% in 2070, making the extension of healthy life expectancy an urgent issue [1]. Sarcopenia is defined as age-related loss of muscle mass and strength. Sarcopenia is a major public health problem that hinders healthy life expectancy in an aging society, particularly as muscle mass has been reported to be associated with physical independence [2] and longevity [3].

Currently, dietary diversity and a protein intake of at least 1.0 g/kg body weight/day are recommended to prevent and improve sarcopenia [4]. In particular, regarding muscle mass, a study of older women showed that the group with the highest protein intake had higher fat-free mass [5]. Similarly, the European Society for Clinical Nutrition and Metabolism (ESPEN) recommends a protein intake of at least 1.0–1.2 g/kg body weight/day for healthy older adults and 1.2–1.5 g/kg body weight/day for older adults with low nutrition or at risk of low nutrition [6]. Furthermore, the relationship between nutrients and muscle mass has been clarified in recent years [7–9]. For example, it has been reported that higher total daily leucine intake is associated with muscle mass and strength in healthy older adults [10].

Against this background, it is recommended that older adults consume protein, especially essential amino acids such as leucine. However, despite known dietary variations related to place of residence, age, and gender, the specific foods and nutrients linked to low muscle mass among urban community-dwelling older adults are not fully understood. Therefore, the purpose of this study was to determine the dietary characteristics of urban community-dwelling older adults with low SMI. This study may allow us to provide more specific nutritional guidance to maintain muscle mass in urban community-dwelling older adults.

## Materials and methods

### Study design and participants

This cross-sectional study used baseline data from the Bunkyo Health Study, a prospective cohort study designed to clarify whether muscle function (muscle mass, muscle strength, and insulin sensitivity) is associated with multiple diseases that necessitate long-term care [11]. Briefly, in this study, we recruited 1,629 older adults aged 65–84 years living in Bunkyo-ku, an urban area in Tokyo, Japan. All participants underwent examinations for 2 days from October 15, 2015, to October 1, 2018. On the first day, we evaluated cognitive function using questionnaires, and also assessed muscle strength, physical performance, and gait speed. On the second day, after an overnight fast, we measured body composition. We excluded three of the 1,629 participants with unavailable data on body composition, and eight of the remaining 1,626 participants because they consumed less than 600 kcal/day (half the energy required for the low physical activity category) or more than 4,000 kcal/day (1.5 times the energy intake required for moderate physical activity category). Finally, we included 1,618 participants (679 males and 939 females) in this analysis. The study protocol was approved by the ethics committee of Juntendo University in November 2015 (Nos. 201,507 and M15-0057). This research was conducted in accordance with the principles outlined in the Declaration of Helsinki. All participants provided written informed consent and were informed that they had the right to withdraw from the trial at any time.

### Skeletal muscle mass measurement

Appendicular skeletal muscle mass (ASM) was evaluated by bioelectrical impedance analysis (InBody770, InBody Japan Inc., Tokyo, Japan). The skeletal muscle mass index (SMI) was calculated by dividing ASM by the square of height (m). Low SMI was defined based on the definition of the Asian Working Group for Sarcopenia (AWGS) 2019 (< 7.0 kg/m^2^ in men and < 5.7 kg/m^2^ in women) [12].

### Dietary assessment

Dietary and nutrient intake were assessed using the brief-type self-administered diet history questionnaire (BDHQ) [13, 14]. The validity of this questionnaire has been reported previously [15]. The BDHQ asks about the frequencies of dietary behaviors and the consumption of 58 foods and beverages over the past month, including the frequencies of daily consumption of 46 foods and nonalcoholic beverage items, rice, and miso soup, the frequency of consumption of five alcoholic beverages and the amount of each alcoholic beverage consumed per drinking occasion, and the frequency of daily consumption of five seasonings (salt, oil, sugar, soy sauce, and noodle soup) used in cooking and the general diet. In this study, in addition to the 58 food items included in the BDHQ, we created a new category for wheat products, defined as bread, ramen, udon, and pasta. Responses to the BDHQ were checked by research staff for completeness, and when necessary, reviewed with participants to ensure that answers were properly understood. Average daily food and nutrient intake was estimated using an ad hoc computer algorithm for the BDHQ, based on Japan’s Standard Tables of Food Composition [15].

### Other measurements

Grip strength was measured twice on each side using a handgrip dynamometer (T.K.K.5401, Takei Scientific Instruments Co., Ltd., Niigata, Japan) [16]. We defined grip strength based on the average of the maximum bilateral values. We administered other physical function tests, including the 10-m walking test to assess maximum gait speed, and the Timed Up and Go test (TUG), which measures the time required for a participant to stand up from a seated position, walk 3 m, turn, walk back, and sit down again. We assessed cognitive function using the Mini-Mental State Examination-Japanese (MMSE-J) [17, 18]. Finally, we evaluated physical activity using the International Physical Activity Questionnaire, which was designed to assess different types of physical activity, such as walking and both moderate- and high-intensity activities.

### Statistical analysis

Participants of each sex were divided into two groups according to the presence or absence of low SMI in each sex. IBM SPSS Statistics for Windows, version 29.0 (IBM Corp., Armonk, NY, USA) was used for the analysis. Data are expressed as medians (interquartile range) or numbers (%). Differences in the characteristics of participants in the low SMI group and the robust group were tested using the Mann-Whitney U test for continuous variables and the chi-squared test for categorical variables. Comparisons of food and nutrient intake between the low SMI group and the robust group were performed using an analysis of covariance adjusted for age, height, weight and physical activity levels. In this analysis, all the food and nutrient intake data showed a non-normal distribution, so we performed a logarithmic transformation prior to analysis. However, for copper and vitamins B1, B2, B6, and B12, which are consumed in trace amounts according to the Japanese Dietary Reference Intakes for 2020, we did not perform a logarithmic transformation [19]. All statistical tests were two-sided with a 5% significance level.

## Results

The characteristics of the study participants are shown in Table 1. The mean participant age was 73.1 ± 5.4 years old. The prevalence of low SMI was 31.1% in men and 43.3% in women. Both men and women in the low SMI group were older than those in the robust group. In addition, both men and women in the low SMI group had significantly lower height, weight, and body mass index (BMI) than those in the robust group. In particular, weight was about 10 kg lower in the low SMI group in both men and women. Furthermore, women in the low SMI group had a significantly lower percentage of body fat than those in the robust group. Regarding physical function, both men and women in the low SMI group had weaker grip strength and slower performance on the TUG test and 10-m walking test than those in the robust group. In men, the MMSE score was significantly lower in the low SMI group than in the robust group.

**Table 1:** Participant characteristics

	Men (*n* = 679)			Women (*n* = 939)		
	Robust	Low SMI	*P*	Robust	Low SMI	*P*
N	468	211		532	407	
Age (years)	71 (68–76)	74 (70–79)	< 0.001	72 (68–76)	74 (69–79)	< 0.001
Height (cm)	167.0 (163.1-170.8)	163.2 (159.7-167.4)	< 0.001	153.7 (150.2-157.5)	151.3 (148.0-154.3)	< 0.001
Weight (kg)	67.1 (62.3–72.6)	57.2 (54.0–61.0)	< 0.001	54.7 (50.7–60.1)	46.5 (43.2–50.0)	< 0.001
BMI (kg/m^2^)	24.3 (22.6–25.9)	21.6 (20.2–22.9)	< 0.001	23.3 (21.6–25.4)	20.3 (18.9–22.0)	< 0.001
SMI (kg/m^2^)	7.6 (7.3–7.9)	6.7 (6.4–6.9)	< 0.001	6.1 (5.9–6.4)	5.4 (5.1–5.5)	< 0.001
Percent body fat (%)	24.6 (20.7–28.4)	24.3 (20.9–28.2)	0.544	32.7 (28.4–37.0)	29.6 (23.8–33.7)	< 0.001
Living alone (%)	54 (11.5)	28 (13.3)	0.301	135 (25.4)	121 (29.7)	0.079
Education (years)	16.0 (12.3–16.0)	16.0 (12.0–16.0)	0.060	12.0 (12.0–14.0)	12.0 (12.0–14.0)	0.210
Brinkmann index	400 (0-800)	400 (0-800)	0.848	0 (0–0)	0 (0–0)	0.845
Sedentary time (hours)	5.0 (4.0–10.0)	5.0 (3.0–8.0)	0.446	5.0 (3.0–7.0)	5.0 (3.5-7.0)	0.390
Physical activity level (METs/hour/week)	33.1 (17.1–61.0)	29.2 (19.8–49.5)	0.269	30.7 (16.5–54.0)	27.1 (14.9–47.6)	0.093
Handgrip strength (kg)	33.7 (30.3–37.0)	29.4 (25.8–32.3)	< 0.001	22.5 (20.4–24.7)	19.7 (17.5–21.7)	< 0.001
Timed Up and Go test score (s)	6.1 (5.5-7.0)	6.6 (5.8–7.4)	< 0.001	6.3 (5.7–7.2)	6.6 (5.9–7.5)	0.004
Gait speed (m/s)	2.0 (1.8–2.2)	1.9 (1.7-2.0)	< 0.001	1.9 (1.7–2.1)	1.8 (1.6-2.0)	< 0.001
MMSE-J (score)	28.0 (27.0–29.0)	27.0 (26.0–29.0)	0.007	28.0 (27.0–29.0)	28.0 (27.0–29.0)	0.076

Data are expressed as medians (interquartile range). The difference between groups was evaluated by the Mann-Whitney U test. BMI: body mass index, MMSE-J: Mini-Mental State Examination-Japanese, SMI: skeletal muscle mass index

Table 2 shows a comparison of nutrient and food intake between the low SMI and robust groups adjusted for age, height, weight and physical activity levels. In women, energy intake was significantly lower in the low SMI group than in the robust group (*p* = 0.002). The intake of protein (*p* = 0.002), fat (*p* = 0.001), and carbohydrates (*p* = 0.015) were also significantly lower in the low SMI group. In contrast, in men, there was no difference in total energy, protein, fat, or carbohydrate intake between the two groups. In women, the intake of almost all nutrients except for alcohol, sodium, sodium chloride equivalent and sucrose was significantly lower in the low SMI group than in the robust group. In men, the low SMI group showed significantly lower intake levels of manganese and Cu compared to the robust group, but no significant differences were observed in other nutrients.

**Table 2:** Comparison of nutrient intake

	Men (*n* = 679)			Women (*n* = 939)		
	Robust	Low SMI	*P*	Robust	Low SMI	*P*
*N*	468	211		532	407	
Energy (kcal/day)	2079.5 (1688.8-2512.5)	2033.0 (1686.0-2359.0)	0.188	1812.0 (1494.8–2239.0)	1754.0 (1429.0-2036.0)	0.002
Protein (g/day)	79.1 (62.3–98.7)	80.5 (61.0-96.4)	0.441	78.1 (61.9-102.2)	77.2 (59.6–94.6)	0.002
Fat (g/day)	60.5 (47.3–77.7)	61.0 (46.7–73.4)	0.749	60.2 (46.5–76.7)	56.0 (43.0-67.7)	0.001
Carbohydrates (g/day)	248.4 (195.1-311.7)	242.2 (200.2-299.7)	0.178	222.1 (180.8-278.3)	212.8 (176.8-261.5)	0.015
Alcohol (g/day)	14.7 (1.0-38.6)	9.5 (1.0-36.2)	0.256	1.0 (1.0-3.7)	1.0 (1.0-2.9)	0.823
Animal protein (g/d)	44.6 (34.0-59.7)	46.4 (33.1–60.0)	0.794	46.9 (33.9–66.4)	46.6 (33.4–60.8)	0.016
Plant protein (g/d)	32.3 (25.8–41.0)	32.0 (26.3–39.3)	0.114	30.8 (24.6–38.2)	29.2 (23.8–36.4)	0.001
Animal fat (g/d)	28.9 (21.9–38.5)	29.1 (21.1–35.9)	0.661	29.8 (21.6–38.7)	27.3 (20.4–35.2)	0.004
Plant fat (g/d)	31.2 (23.1–39.1)	31.8 (24.2–39.8)	0.747	30.2 (22.9–37.3)	28.0 (22.0-34.2)	0.003
Ash (g/d)	21.4 (17.6–26.9)	22.2 (17.3–26.1)	0.373	21.1 (16.9–26.9)	20.8 (16.9–24.7)	0.011
Sodium (mg/d)	5033.0 (4107.6-6165.3)	4929.4 (4143.0-6090.5)	0.622	4521.2 (3704.8-5866.8)	4464.2 (3687.4-5442.5)	0.110
Potassium (mg/d)	2913.9 (2301.8-3791.1)	2964.5 (2240.7-3695.8)	0.195	3252.0 (2417.6-4188.8)	3013.6 (2346.0-3809.0)	< 0.001
Calcium (mg/d)	631.8 (485.7-838.2)	640.9 (479.5-834.3)	0.399	693.3 (512.0-891.1)	666.8 (506.1-841.4)	0.011
Magnesium (mg/d)	291.1 (232.2-377.6)	305.6 (231.3-357.8)	0.220	302.0 (232.6-383.8)	290.3 (226.1-356.5)	0.001
Pi (mg/d)	1199.6 (950.4-1525.8)	1246.3 (919.4-1475.8)	0.375	1220.2 (960.7-1570.9)	1197.9 (935.2-1447.1)	0.002
Fe (mg/d)	9.1 (7.1–11.8)	9.3 (6.9–11.2)	0.133	9.6 (7.5–12.3)	9.3 (7.2–11.5)	< 0.001
Retinol (µg/d)	460.3 (261.2-749.6)	429.2 (260.8-721.4)	0.489	381.8 (267.6-600.2)	341.4 (242.0-625.5)	0.019
β-carotene (µg/d)	3626.2 (2206.5-5577.7)	3618.9 (1987.6-5812.6)	0.051	4750.2 (3011.6–7144.0)	4646.5 (2915.8-6295.8)	0.016
Zinc (mg/d)	8.9 (7.1–11.0)	9.0 (6.7–10.7)	0.141	8.8 (7.1–11.3)	8.5 (6.8–10.3)	< 0.001
Manganese (mg/d)	3.4 (2.6–4.4)	3.4 (2.6–4.2)	0.013	3.5 (2.7–4.3)	3.4 (2.7–4.3)	0.001
Retinol equivalent (µg/d)	851.0 (539.2-1161.4)	812.2 (563.2-1111.5)	0.252	839.8 (604.8-1145.9)	789.5 (531.2-1133.7)	0.001
Vitamin D (µg/d)	15.1 (9.3–23.0)	15.3 (9.3–23.3)	0.835	17.2 (10.1–28.1)	17.1 (10.1–25.9)	0.037
α-tocopherol (mg/d)	8.3 (6.4–10.7)	8.6 (6.4–10.8)	0.746	9.0 (6.7–11.7)	8.4 (6.7–10.5)	0.002
Vitamin K (µg/d)	369.8 (237.6-516.9)	356.3 (212.7-514.8)	0.135	405.1 (270.7-546.1)	367.6 (245.3-530.3)	0.003
Niacin (mg/d)	19.0 (15.2–25.1)	19.2 (14.5–23.9)	0.446	19.3 (14.9–26.5)	18.4 (14.4–23.4)	0.001
Folic acid (µg/d)	390.3 (296.0-514.2)	398.1 (285.0-511.5)	0.091	427.7 (332.4-575.6)	417.4 (321.0-530.6)	0.001
Pantothenic acid (mg/d)	7.4 (6.0-9.3)	7.4 (5.7–9.1)	0.254	7.6 (5.9–9.5)	7.2 (5.7-9.0)	< 0.001
Vitamin C (mg/d)	127.7 (91.8-176.7)	133.6 (89.8-185.1)	0.415	159.4 (111.8-225.1)	147.7 (114.4–205.0)	0.025
Saturated fatty acids (g/d)	16.2 (12.4–21.5)	16.2 (11.5–19.8)	0.621	16.1 (12.4–20.8)	15.0 (11.4–18.5)	0.003
Monounsaturated fatty acids (g/d)	21.3 (16.6–27.6)	21.6 (16.2–26.6)	0.783	21.2 (16.3–26.9)	19.5 (14.7–24.3)	0.001
Polyunsaturated fatty acids (g/d)	14.8 (11.3–18.7)	14.4 (11.5–18.5)	0.900	14.1 (10.9–18.2)	13.6 (10.3–16.1)	0.900
n-3 fatty acids (g/d)	3.1 (2.3–4.1)	3.1 (2.2-4.0)	0.980	3.0 (2.2–4.2)	2.8 (2.2–3.8)	0.003
n-6 fatty acids (g/d)	11.5 (8.8–14.5)	11.3 (8.9–14.4)	0.870	10.9 (8.6–14.0)	10.2 (8.1–12.4)	0.002
Cholesterol (mg/d)	434.6 (300.7-578.1)	419.4 (290.3–568.0)	0.709	435.4 (316.9-588.4)	441.9 (304.9-570.2)	0.046
Soluble fiber (g/d)	3.6 (2.6–4.8)	3.6 (2.5–4.7)	0.165	3.9 (2.9–5.3)	3.7 (2.8–4.8)	< 0.001
Insoluble fiber (g/d)	9.9 (7.5–13.0)	10.1 (7.6–12.9)	0.084	10.8 (8.1–14.2)	10.3 (8.0–13.0)	0.001
Total dietary fiber (g/d)	14.0 (10.5–18.5)	14.3 (10.2–18.1)	0.088	15.3 (11.5–20.2)	14.5 (11.1–18.3)	0.001
Sodium chloride equivalents (g/d)	12.7 (10.4–15.5)	12.4 (10.5–15.4)	0.645	11.4 (9.4–14.8)	11.2 (9.3–13.7)	0.114
Sucrose (g/d)	11.8 (7.1–19.8)	13.3 (7.7–20.4)	0.731	13.1 (7.8–20.2)	12.7 (7.4–19.7)	0.148
Daidzein (mg/d)	14.4 (8.0-25.8)	14.7 (7.8–22.7)	0.339	15.9 (9.0-24.5)	14.6 (8.3–22.9)	0.003
Genistein (mg/d)	24.4 (13.5–43.3)	25.2 (13.1–38.1)	0.348	27.1 (15.3–41.4)	24.8 (14.1–38.8)	0.003
Vitamin B1 (mg/d)	0.9 (0.7–1.1)	0.9 (0.7–1.1)	0.146	0.9 (0.7–1.2)	0.9 (0.7–1.1)	< 0.001
Vitamin B2 (mg/d)	1.5 (1.2-2.0)	1.5 (1.2–1.9)	0.134	1.6 (1.3-2.0)	1.5 (1.2–1.9)	< 0.001
Vitamin B6 (mg/d)	1.5 (1.1–1.9)	1.5 (1.1–1.8)	0.141	1.6 (1.2-2.0)	1.5 (1.2–1.8)	< 0.001
Vitamin B12 (µg/d)	10.9 (7.4–16.2)	10.9 (7.1–16.1)	0.642	11.4 (7.2–17.2)	10.6 (7.2–15.8)	0.010
Cu (mg/d)	1.3 (1.0-1.6)	1.3 (1.0-1.5)	0.034	1.3 (1.0-1.6)	1.2 (1.0-1.5)	< 0.001

Data are expressed as medians (interquartile range). Data adjusted for age, height, weight and physical activity levels. SMI: skeletal muscle mass index

In terms of food intake (Table 3), all food intake was similar between the low SMI group and the robust group, in men. In women, the intake of high-fat fish and tofu or fried tofu was higher in the robust group than in the low SMI group, and the intake of pasta and wheat products was higher in the low SMI group than in the robust group. There were no significant differences in the intake of other foods, including vegetables and beverages, in either men or women (data not shown).

**Table 3:** Comparison of food intake (carbohydrate and protein intake)

	Men (*n* = 679)			Women (*n* = 939)		
	Robust	Low SMI	*P*	Robust	Low SMI	*P*
N	468	211		532	407	
Bread (g/day)	57.7 (23.1–72.8)	64.7 (28.9–72.8)	0.452	56.0 (22.5–63.0)	56.0 (22.5–63.0)	0.510
Soba (g/day)	20.8 (9.7–52.0)	20.8 (10.8–52.0)	0.899	9.3 (7.5–20.0)	9.3 (1.0–20.0)	0.952
Udon (g/day)	20.8 (9.7–46.2)	20.8 (9.7–52.0)	0.707	16.0 (7.5–23.5)	16.0 (7.5–40.0)	0.133
Ramen (g/day)	10.8 (8.6–23.1)	10.8 (1.0-23.1)	0.551	7.5 (1.0-10.1)	7.5 (1.0-9.3)	0.873
Pasta (g/day)	10.8 (8.6–20.8)	10.8 (8.6–20.8)	0.906	8.4 (1.0–16.0)	8.4 (1.0–16.0)	0.018
Wheat products (g/day)	103.9 (72.8-145.5)	119.5 (81.9-166.3)	0.336	81.0 (54.9-116.4)	83.7 (58.5–112.0)	0.047
Rice (g/day)	240.2 (135.1-300.3)	180.2 (135.1-300.3)	0.076	143.0 (104.0-260.0)	130.0 (104.0-234.0)	0.147
Potato (g/day)	45.0 (18.0-63.5)	50.0 (20.0-69.3)	0.641	45.0 (20.0-69.3)	45.0 (20.0-69.3)	0.969
Milk (g/day)	135.0 (11.1-173.2)	107.1 (1.0-165.0)	0.167	107.1 (8.3–165.0)	123.7 (10.0-173.2)	0.646
Chicken (g/day)	17.1 (11.1–35.6)	15.7 (7.3–35.6)	0.146	17.1 (11.1–35.6)	15.7 (8.0-33.9)	0.655
Pork/beef (g/day)	35.6 (24.6–60.1)	33.9 (14.8–39.1)	0.268	35.6 (27.7–61.6)	33.9 (17.1–42.7)	0.161
Ham (g/day)	5.9 (2.5–14.8)	5.1 (2.3–13.6)	0.810	8.6 (2.4–14.8)	5.4 (2.4–13.6)	0.096
Liver (g/day)	1.0 (1.0-2.9)	1.0 (1.0-2.7)	0.165	1.0 (1.0-2.9)	1.0 (1.0-2.9)	0.338
Squid/octopus/shrimp/shellfish (g/day)	13.1 (6.7–18.2)	8.5 (6.1–16.7)	0.847	13.1 (6.7–18.2)	11.8 (6.7–18.2)	0.658
Fish with bone (g/day)	6.4 (4.1–19.7)	5.5 (1.0-22.2)	0.791	5.8 (1.0-22.2)	5.8 (4.1–13.7)	0.900
Tuna can (g/day)	2.8 (1.0-3.9)	3.1 (1.0-4.2)	0.118	2.7 (1.0-3.9)	2.8 (1.0-3.9)	0.719
Dried fish (g/day)	15.2 (10.3–33.0)	13.7 (6.8–33.0)	0.853	14.5 (9.1–33.0)	14.5 (10.3–33.0)	0.774
High-fat fish (g/day)	15.2 (7.8–36.1)	15.2 (7.4–36.1)	0.612	15.8 (10.5–36.1)	15.2 (7.8–34.2)	0.043
Low-fat fish (g/day)	14.5 (7.5–36.1)	15.2 (7.8–32.9)	0.933	15.2 (7.8–32.9)	14.5 (7.4–32.9)	0.706
Egg (g/day)	37.7 (23.6–66.0)	42.4 (21.2–72.6)	0.955	42.4 (23.6–68.6)	32.7 (23.6–66.0)	0.570
Tofu/fried tofu (g/day)	41.9 (21.5–78.2)	48.4 (31.4–97.7)	0.053	44.3 (31.4–83.8)	40.3 (17.7–83.8)	0.024
Natto (g/day)	16.1 (4.2–35.4)	18.6 (3.6–40.8)	0.558	16.7 (3.8–36.0)	16.1 (5.8–36.0)	0.539

Data are expressed as medians (interquartile range). Data are adjusted for age, height, weight and physical activity levels. SMI: skeletal muscle mass index

## Discussion

This study examined the nutritional and food intake characteristics of urban community-dwelling older adults with low muscle mass. In men, there were no significant differences in food intake, including total energy intake, between the low SMI group and the robust group. Conversely, in women, the low SMI group had less total energy intake, consumed lower amounts of energy-producing nutrients (protein, fat, and carbohydrates), high-fat fish and tofu/fried tofu than the robust group, but the low SMI group consumed significantly more pasta and wheat products than the robust group.

In the present study, there were sex-related dietary differences between robust older adults and those with low muscle mass. Sex differences have been demonstrated in nutritional behavior, styles of nutrition, dietary profiles, approach to nourishment, approach to the location of meal consumption, and the sources of nutritional knowledge; women in particular have greater trust in the benefits of healthy nutrition, are more active in controlling their weight, and choose healthier foods than men [20]. In addition, women may differ from men in their eating behaviors, regardless of age [21], and sex differences have been observed in the perception of food [20]. In this study, total energy intake and the intake of almost all nutrients were lower in the low SMI group than in the robust group in women, but there were few differences in terms of the actual foods consumed. It is suggesting that there were few differences in dietary balance and only small differences in each food intake. This suggests that women need nutritional guidance to increase their overall dietary intake and thereby prevent sarcopenia. Regarding high-fat fish, it’s worth noting that its intake was significantly lower in the low SMI group than in the robust group. High-fat fish contain n-3 fatty acids, whose intake has been reported to have a variety of beneficial effects, including preventing cardiovascular disease [22], lowering blood pressure [23], and lowering triglycerides [24]. A 6-month randomized controlled trial showed that compared to controls, subjects who received n-3 polyunsaturated fatty acids for 6 months exhibited significantly increased thigh muscle volume, handgrip strength, and one-repetition maximum strength, suggesting an effect on improving physical performance in older adults [25].

In men, there were no significant differences in food intake, including total energy intake, between the low SMI group and the robust group. However, although not statistically significant, the low SMI group tended to consume more bread and other wheat products and less rice in the carbohydrate component compared to the robust group. In addition, the low SMI group tended to consume less animal protein, including dairy products, and more plant protein than the robust group in the protein component. A survey on diet and lifestyle conducted by the Ministry of Agriculture, Forestry, and Fisheries (MAFF) reported that the older the age of Japanese people, the more bread they tend to consume as part of their staple food intake [26]. Bread requires fewer cooking steps than rice and noodles, and there is a variety of breads, such as sandwiches and sweet breads, to provide satisfaction in a single meal. This may therefore lower the hurdle to eating and make it more feasible for older adults, whose oral functions are declining. For similar reasons, it is possible that men in the low SMI group consumed higher amounts of tofu or fried tofu compared to those in the robust group. In addition, animal proteins are rich in branched-chain amino acids, which are necessary to maintain muscle mass, and a previous study in older women reported that a high intake of animal protein was significantly associated with fat-free mass [5]. Further, a meta-analysis and systematic review in middle-aged and older adults showed that dairy protein intake was associated with a significant increase in muscle mass, suggesting that dairy products are important for maintaining muscle mass [27]. Thus, protein, especially animal protein, is needed for the prevention of muscle mass loss in older adults. However, studies have shown a gender disadvantage for men compared to women in nutrition education [21]. Thus, men may need detailed guidance on specific foods to prevent sarcopenia.

A previous study reported that older adults at low risk of sarcopenia and frailty intake more energy, nutrients, and foods than older adults at high risk [28]. Another study showed that even after adjusting for age, lifestyle, physical activity, and energy intake, older adults with high SMI consumed more vegetables than those with low SMI [7]. Other studies have shown that older adults with higher dietary quality and dietary diversity have a lower risk of sarcopenia and frailty [29–31], and fruits and vegetables in particular have been noted to reduce these risks [32, 33]. In this study, there were no significant differences in the intake of vegetables and fruit between the low SMI and robust groups in either men or women. The National Health and Nutrition Survey showed that older adults consumed more fruits and vegetables than other age groups [34]. A recent survey reported that the average vegetable intake of older adults was 288.3 g/day in men and 273.6 g/day in women [34]. In this study, the average vegetable intake in men was 276.2 g/day in the robust group and 273.3 g/day in the low SMI group, while in women it was 268.8 g/day in the robust group and 265.82 g/day in the low SMI group; thus both groups in both sexes consumed a roughly average amount of vegetables. In addition, while a survey of fruit intake in older adults showed that approximately 40% of the population consumed less than 100 g/day [34], men in this study consumed 109.3 g/day and women consumed 110.3 g/day, suggesting that this study population is also characterized by a near-average amount of fruit intake. Thus, among urban community-dwelling older adults who consume some fruits and vegetables, their intake is not associated with low SMI, but the reason for this is unknown.

In this study, both muscle mass and body weight were about 15% lower in the low SMI group than in the robust group in men and women alike. On the other hand, percent body fat and dietary intake showed little difference between the groups. This may be due not only to food intake but also to other factors such as digestion, absorption, anabolism, and catabolism. The production of digestive enzymes generally decline with age, resulting in reduced absorption of several nutrients [35]. In addition, aging often leads to a decrease in anabolic signals and an increase in catabolic signals, contributing to muscle loss (sarcopenia) and other age-related changes [36]. This could be exacerbated by factors such as increased cytokine levels, reduced dietary energy intake, insulin resistance, and decreased growth hormone-insulin-like growth factor-I concentrations. Further, the intestinal microflora is associated with poor digestion and absorption in older adults, and this can contribute to malnutrition and other health issues. Changes in lifestyle, diet, and possibly the use of prebiotics and probiotics may help to mitigate these effects [37, 38]. Therefore, important factors for maintaining muscle mass include not only energy intake but also the management of digestion, absorption, and chronic diseases. In addition, both men and women with low SMI may need more energy intake to increase skeletal muscle mass.

There are several limitations to this study. First, the BDHQ was used for the dietary survey in this study. It is possible that some nutrients and foods were not included by the BDHQ used in this dietary survey. In addition, the study was conducted in older adults, and recall bias could not be fully eliminated. The frequency of under- and over-reporting in dietary surveys has been found to vary according to the characteristics of the subject [39, 40], and there are no reports on issues related to reporting by older adults with low muscle mass, sarcopenia, and frailty. In addition, the times at which food was eaten by the study participants were unknown. A recent study showed that protein intake at breakfast was positively correlated with muscle function in older women [41], and in particular that higher quality protein consumed at breakfast was associated with reduced muscle weakness [42]. Therefore, the timing of food intake and the specific foods eaten should be considered. The second limitation involves the subject characteristics. The study subjects were older adults living in urban areas, who may have a high educational background and high levels of health literacy. Therefore, further research is needed to generalize these results. Next, this study may be prone to Type I errors due to multiple testing. When testing differences for multiple items simultaneously, there is a risk of false positives when using a significance threshold of 0.05. Therefore, some findings, such as those shown in Table 3, should be interpreted with caution because of the potential for inflated Type I errors. Finally, as this was a cross-sectional study, it is difficult to establish causal relationships.

In conclusion, there were sex differences in dietary characteristics between urban community-dwelling older adults with low SMI and those who were robust. This suggests that dietary guidance for the prevention of muscle loss and sarcopenia in older adults should consider sex differences. Advising women to increase their total energy intake may be important in preventing muscle loss. For men, further study is needed, as there were no significant differences observed in the intake of almost all food items, including total energy intake, between the low SMI group and the robust group.

## Data Availability

Some or all datasets generated and/or analyzed during the current study are not publicly available; however they can be obtained from the corresponding author upon a reasonable request.

## Abbreviations

SMI: Skeletal Muscle mass Index
ESPEN: European Society for Clinical Nutrition and Metabolism
ASM: Appendicular skeletal muscle mass
AWGS: Asian Working Group for Sarcopenia
BDHQ: Brief-type self-administered Diet History Questionnaire
TUG: Timed Up and Go test
MMSE-J: Mini-Mental State Examination-Japanese
BMI: Body mass index
MAFF: Ministry of Agriculture, Forestry, and Fisheries
